# A comparison of head movements tests in force plate and accelerometer based posturography in patients with balance problems due to vestibular dysfunction

**DOI:** 10.1038/s41598-021-98695-1

**Published:** 2021-09-27

**Authors:** Magdalena Janc, Mariola Sliwinska-Kowalska, Magdalena Jozefowicz-Korczynska, Pawel Marciniak, Oskar Rosiak, Rafal Kotas, Zuzanna Szmytke, Joanna Grodecka, Ewa Zamyslowska-Szmytke

**Affiliations:** 1 Audiology and Phoniatrics Clinic, Nofer Institute of Occupational Medicine, 8 St Therese Str., 91-348 Lodz, Poland; 2 Balance Disorders Unit, Department of Otolaryngology, The Norbert Barlicki Memorial Teaching Hospital, Medical University of Lodz, Lodz, Poland; 3 Department of Microelectronics and Computer Science, Lodz University of Technology, Lodz, Poland; 4 Lodz University of Technology, Lodz, Poland

**Keywords:** Physical examination, Computer science

## Abstract

This study compares HS posturography on inertial sensors (MediPost) with force platform posturography in patients with unilateral vestibular dysfunction. The study group included 38 patients (age 50.6; SD 11.6) with unilateral vestibular weakness (UV) and 65 healthy volunteers (48.7; SD 11.5). HS tests were performed simultaneously on the force plate and with MediPost sensor attached at L4. Four conditions applied: eyes open/closed, firm/foam. The tests were performed twice, with the head moving at the frequency of 0.3 Hz (HS 0.3) and 0.6 Hz (HS 0.6). Mean sway velocity was significantly lower for MediPost than force plate in 4th condition both in UV and healthy group. For HS 0.3 the differences between devices were marginal; the highest sensitivity (87%) and specificity (95%) were in 4th condition. For HS 0.6 MediPost revealed lower sensitivity than force plate although the surface parameter improved results. MediPost IMU device and force platform posturography revealed a similar ability to differentiate between patients with balance problems in course of vestibular pathology and healthy participants, despite the differences observed between measuring methods. In some tests surface parameter may be more appropriate than sway velocity in improving MediPost sensitivity.

## Introduction

The head shaking (HS) posturography is a relatively new method, in which we hypothesize that horizontal head movements elicit balance perturbations in patients with a peripheral vestibular deficit. Pilot studies on dynamic posturography performed in 1998 by Shepard and Speers revealed an increased body sway in the patients with a peripheral vestibular dysfunction and normal results for the dynamic posturography without head movements^1^. Further research revealed up to 70% sensitivity and 100% specificity of the 5th condition of the dynamic posturography, with the slow head movements (15°/s) differentiating subjects with and without vestibular impairment^2^. For comparison, the sensitivity and specificity of the dynamic posturography without head movements are 40% and 90%, respectively^3^.

The concept of head movements in static posturography was introduced by Cohen et al. in a 2014 study of a clinically heterogenous group with vestibular diseases^4^. They used slow head movements of 0.33 Hz and assessed such parameters as: trial duration (seconds), number of head movements during pitch and jaw head movements measured with head mounted inertial sensors (inertial measurement unit—IMU) and kinematic measures derived from a torso-mounted IMU. The data was then computed to quantify the sway in the mediolateral and anteroposterior directions for the trunk. However, the torso IMU data was only used if the head mounted IMU data indicated that the subject was able to move their head in at least 5 cycles during the trial. According to Cohen, 75% of subjects in age group of 60–79 years were not able to remain standing for 10 s (mean time was 5.2 s)^5^. In regard to adding the head movements to the standard protocol of the Modified Clinical Test of Sensory Interaction in Balance (mCTSiB), there have been no studies on the topic until our previous paper^6^. In this study, the sensitivity and the specificity of the 4th condition (eyes closed, foam) of the HS static posturography were 74% and 71% in the group of patients with a vestibular neuritis.

Until recently, the only objective measure of sways in patients with balance disorders was tensometric posturography. However, a posturography based on accelerometers and gyroscopes mounted to the human body near the center of mass has become more popular^7,8^. The new method of inertial sensors measurements seems to be cheaper, more comfortable and gives the opportunity to assess the balance in circumstances similar to everyday movements^9,10^.

The main problem was the tensiometer platform and the accelerometers posturography applying different measurement principles, which may interfere with the diagnostic process’ integrity. For people with balance disorders, the reliability of the sensor-based sways recording method was strongly related to measurements on the force plate under static conditions^9,11^. However, the differences between the tools may appear to be significant when considering the head movement conditions.

The aim of this study was to compare the results of head shaking posturography performed in accordance with the same tasks protocol on the MediPost IMU device and the standard force platform posturography. The main concern is how the differences between devices, if present, are clinically relevant in patients with unilateral vestibular weakness, the second point of concern being whether the sway velocity is the most appropriate measuring parameter.

## Materials and methods

The study group included 38 patients diagnosed in the Audiology Clinic due to vertigo and dizziness. The participants’ age varied from 28 to 71 years (mean age 50.6; SD 11.6 years).

Diagnostic evaluation of vestibular system was based on a combination of symptoms, physical examination of the vestibular, ocular motor and cerebellar systems, caloric test, and rotational chair tests. The diagnostic procedure has been simplified by using the Bárány Society classification of vestibular disorders^12^. Additionally, the symptom’s intensity and handicap were evaluated basing on the Dizziness Handicap Inventory (DHI) and the Vestibular Symptom Scale (VSS).

The inclusion criteria for the study group included:

- Unilateral vestibular impairment in the caloric test and vestibular and balance symptoms reported in the questionnaires.
- Abnormal Equilibrium Score or vestibular subscale in dynamic posturography.

Only the patients with a non-compensated peripheral unilateral vestibular weakness and balance problems were included in the study group (UV group). The asymmetry of the vestibular response was confirmed in the videonystagmography (VNG) bi-thermal (44° and 30 °C) water caloric test. The maximal slow phase velocity (SPV, °/s) was measured, and the percentage of response asymmetry (canal paresis, CP) has been calculated by a computer software. A canal paresis (CP) equal to or greater than 30% was considered asymmetrical. CP was considered normal, if the difference between both ears was less than 30%, to exclude the borderline values, which is in agreement with several literature findings^13^. The balance was assessed by the Computerized Dynamic Posturography (Neurocom), in which the equilibrium score (ES) was calculated by the software as a general balance measurement. A vestibular subscale was also considered.

The patients were also tested with the VNG oculomotor tests: saccades, smooth pursuit and optokinetic in order to assess central abnormalities of the balance system. Patients showing any signs of the central abnormalities were excluded from the study.

Finally, the study group (UV) included patients with vestibular neuritis, ones who underwent gamma-knife surgery due to unilateral vestibular nerve schwannoma autoimmunological ear disorder and ones with unilateral peripheral vestibular impairment of unknown etiology, all in the subacute and chronic stage at least 2 weeks from onset or with a definite Meniere disease^14^. Six patients (16%) self-reported anxiety or depression apart from the main diagnosis. Sixteen patients (42%) reported a high level of disability, measured with Dizziness Handicap Inventory (> 54p), 34 patients (90%) reported vestibular symptoms in accordance with the Vestibular Symptom Scale (VSS).

The reference group (H group) included 65 healthy participants aged from 20 to 74 years (mean age 48.7; SD 11.5 years), who were recruited voluntarily. The inclusion criteria for H group were the following: no history of vertigo, dizziness, imbalance, known neurologic, circulatory and musculoskeletal system disorders, diabetes, or migraine. The clinical history was then verified in the physical examination, the VNG testing (with bi-thermal water caloric test) with sinusoidal rotary chair and the dynamic posturography. The physical examination and testing were performed according to the exact same protocol as in the study group and any abnormal results excluded the volunteer from further participation in the study.

The research protocol was approved by the Bioethics Commission (17/2014). All subjects provided written informed consent. All clinical investigation was conducted according to the principles included in the Declaration of Helsinki.

### Test protocol

**Figure 1:**
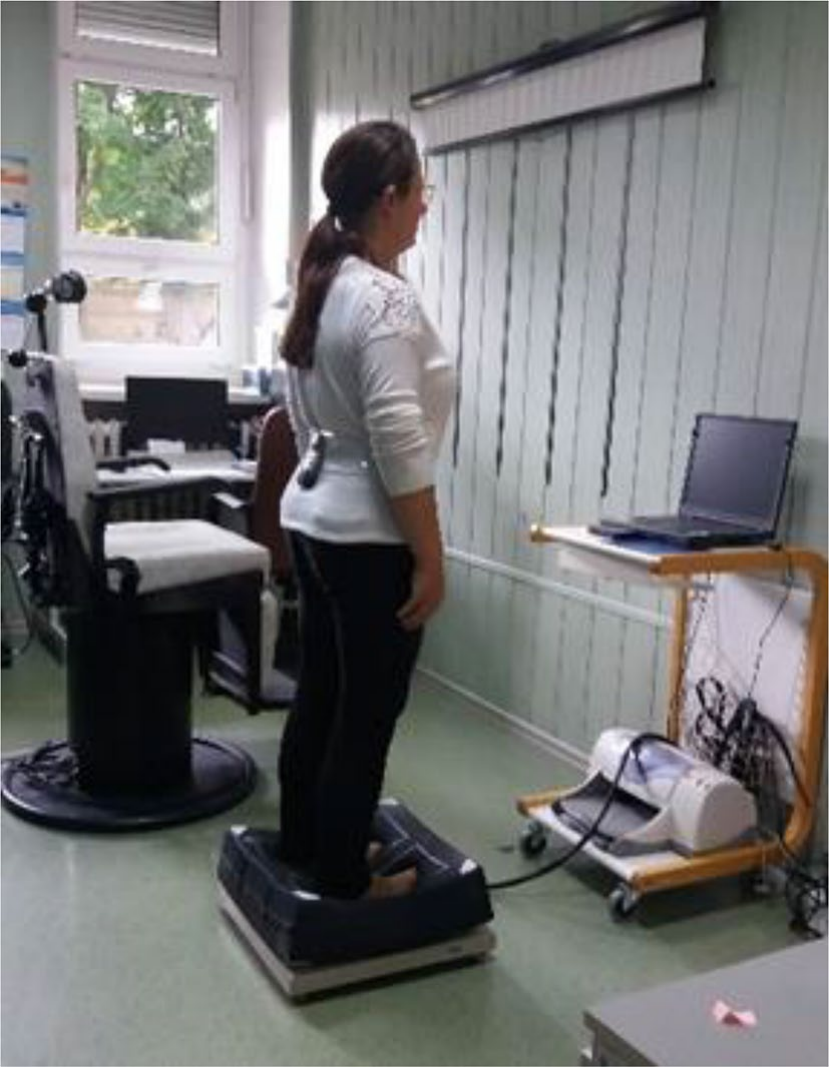
The 4th trial of mCTSIB protocol with head movements, which was simultaneously recorded on MediPost and the platform.

A Modified Clinical Test of Sensory Interaction on Balance (mCTSIB) protocol was used in this study, which was conducted on the NeuroCom^®^ Static Balance Master posturography (P) equipment. The mCTSIB was recorded simultaneously with the inertial measurement unit (IMU) device (MediPost) attached at the lumbar level (Fig. 1).

In the mCTSIB protocol, the subjects were asked to stand with their hands at their sides, feet apart, and perform the following four sensory conditions: standing on a firm (1st condition) and foam (3rd) surface with eyes open; standing on a firm (2nd) and foam (4th) surface with eyes closed (SP). Every condition included three repetitions lasting 10 s, which means 30 s per condition. The mean value was calculated from the repetitions as one value per condition.

The tests were performed twice, with the head moving at the frequency of 0.3 Hz (HS 0.3), 15˚ to the right and to the left from the central position and at the frequency of 0.6 Hz (HS 0.6) in the same range. Before testing, the patient was taught the rhythm and proper range of the movement by a technician. During the test patient controlled the movement by listening to a metronome beat and the technician observed and controlled the range of head movement from the left to the right shoulder. The order of the tests’ frequency was random. The foam dimensions were the following: 45 × 45 × 12.3 cm, with density of 64 kg/m^3^.

If the subject was not able to maintain balance during the test, their fall was automatically marked by the platform posturography software, and the value of 6˚/s was recorded in the result sheet. The same principle was applied for MediPost.

### Data analysis

The MediPost (M) prototype is a wearable, battery powered, lightweight device controlled by a ESP32 system with a Wi-Fi radio unit. It relies on a 3-axis inertial measurement unit (IMU) to determine its orientation in space. The IMU (STMicroelectronics LSM9DS1) contains a micro-electro-mechanical system (MEMS) consisting of an accelerometer, a gyroscope, and a magnetometer. The device is synchronized and controlled by a computer program. The application connects to a predefined Wi-Fi network and then starts to download data from the MediPost via a defined port. Sampling frequency of 200 Hz is used on the IMU device. Then a low-pass filter is also implemented on the IMU, after which the signal can be represented with 20 samples per second. There are two reasons for such a solution: a decrease in the energy consumption caused by the data transmission over the Wi-Fi protocol and an increase of the calculation speed in the analysis software designed for the system. The data package is collected 20 times per second and consists of all information taken from the three sensors of the IMU, in the three axes (9 values in total). To determine the angular position of the device, the IMU Madgwick algorithm was chosen. The operation of this algorithm is based on the use of quaternions for the representation of orientation in 3D space. A detailed description of the calculation of the parameters was presented in the previous study^15^.

**Figure 2:**
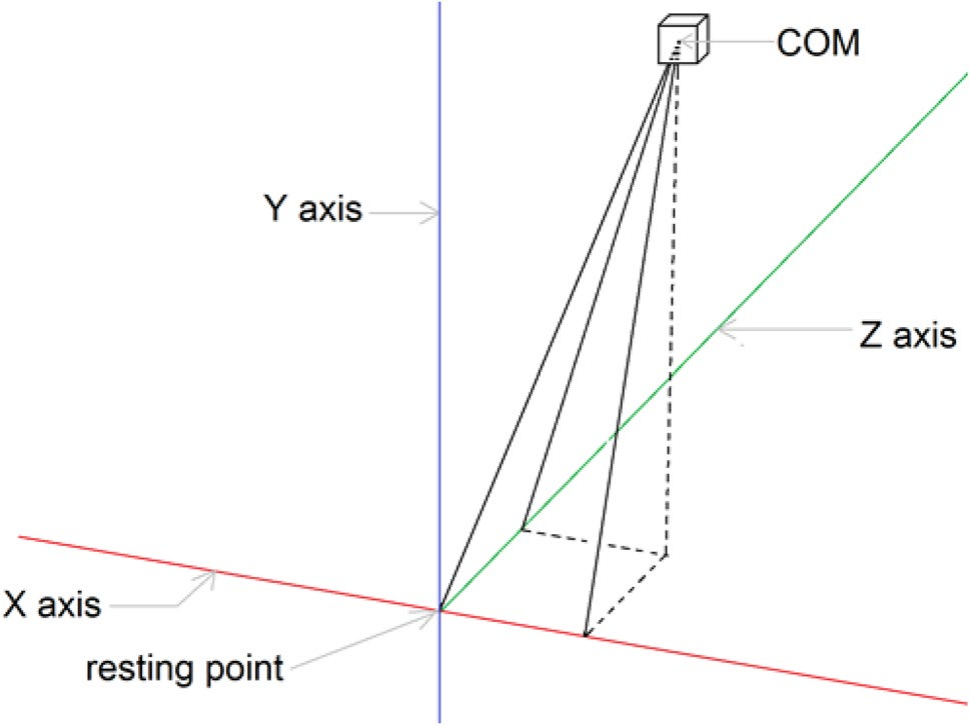
Determining the coordinates of the projection of the COM (a simplified 3D scheme).

Figure 2 presents a simplified scheme showing the principles for determining the coordinates of the projection of the COG.

The designed system enables calculations of the following parameters:

- Current angular speed resulting from the roll and pitch rotations:
  1 $$ \omega = \frac{{{\text{hypot}}(pitch_{i} - pitch_{i - 1} ,roll_{i} - roll_{i - 1} )}}{T} $$

where:

2 $$ hypot(x,y) = \sqrt {x^{2} + y^{2} } $$

and:

the pitch and roll are angles, as shown in Fig. 2, *i* is the time-step number.

- Maximum angular speed resulting from the roll and pitch rotations.
- Average angular speed resulting from the roll and pitch rotations.
- The trajectory length:
  3 $${\sum }_{i=1}^{N}hypot\left({x}_{i}-{x}_{i-1},{y}_{i}-{y}_{i-1}\right)$$
  where *N* is the number of samples and *i* is the time-step number.
- The surface area:
  4 $${\sum }_{i=1}^{N}\left|\left(\left({x}_{i-1}-{x}_{0}\right)\left({y}_{i}-{y}_{0}\right)-\left({x}_{i}-{x}_{0}\right)\left({y}_{i-1}-{y}_{0}\right)\right)\right|$$

where *N* is the number of samples, *i* is the time-step number, *x*_*0*_ and *y*_*0*_ are the coordinates of the geometric center of the trajectory (resting point).

The angular sway velocity [°/s] was the main outcome from both the platform posturography and MediPost. The measurements results illustrating the relationship between Platform (P) and MediPost (M) were presented on a Bland–Altman plot and were calculated with Spearman’s correlation coefficients with moderate 0.4–0.69, strong 0.7–0.89, and very strong 0.9–1 ranges^16^.

Preliminary data analysis allowed to determine the distribution of the obtained results. The histogram and box plot were used to verify the distribution type. These two techniques allowed to determine the frequency of each value in the data set and the 5-number summary of variable: minimum, first quartile, median, third quartile and maximum. On this basis it was concluded that the collected data is not normally distributed. For this reason, a Non-parametric Wilcoxon paired test was used to compare the mean angular velocity [˚/s] values between the posturography and the MediPost results separately in both the UV and the healthy groups.

The Receiver Operating Characteristic (ROC) curves were used to establish the sensitivity and specificity of every condition. The area under the ROC curve (AUC) was calculated, while the statistical significance was established at 0.05. The cut-off point was calculated with the Youden Index.

Only in the MediPost the ROC curves were calculated for angular velocity [˚/s], the length of trajectory [mm] and the surface of COM alignment [mm^2^].

The criterion of statistical significance p < 0.05 was used in the statistical analyses. IBM SPSS Statistics 22 was used.

### Declarations

All procedures performed in studies involving human participants were in accordance with the ethical standards of the institutional and/or national research committee and with the 1964 Helsinki Declaration and its later amendments, or comparable ethical standards. The study was approved by the Bioethics Committee of the Nofer Institute of Occupational Medicine, Lodz, Poland (No of protocol 17/2014).

## Results

1. The comparison of mean values of the tests results between the platform posturography and the IMU MediPost device.

**Figure 3:**
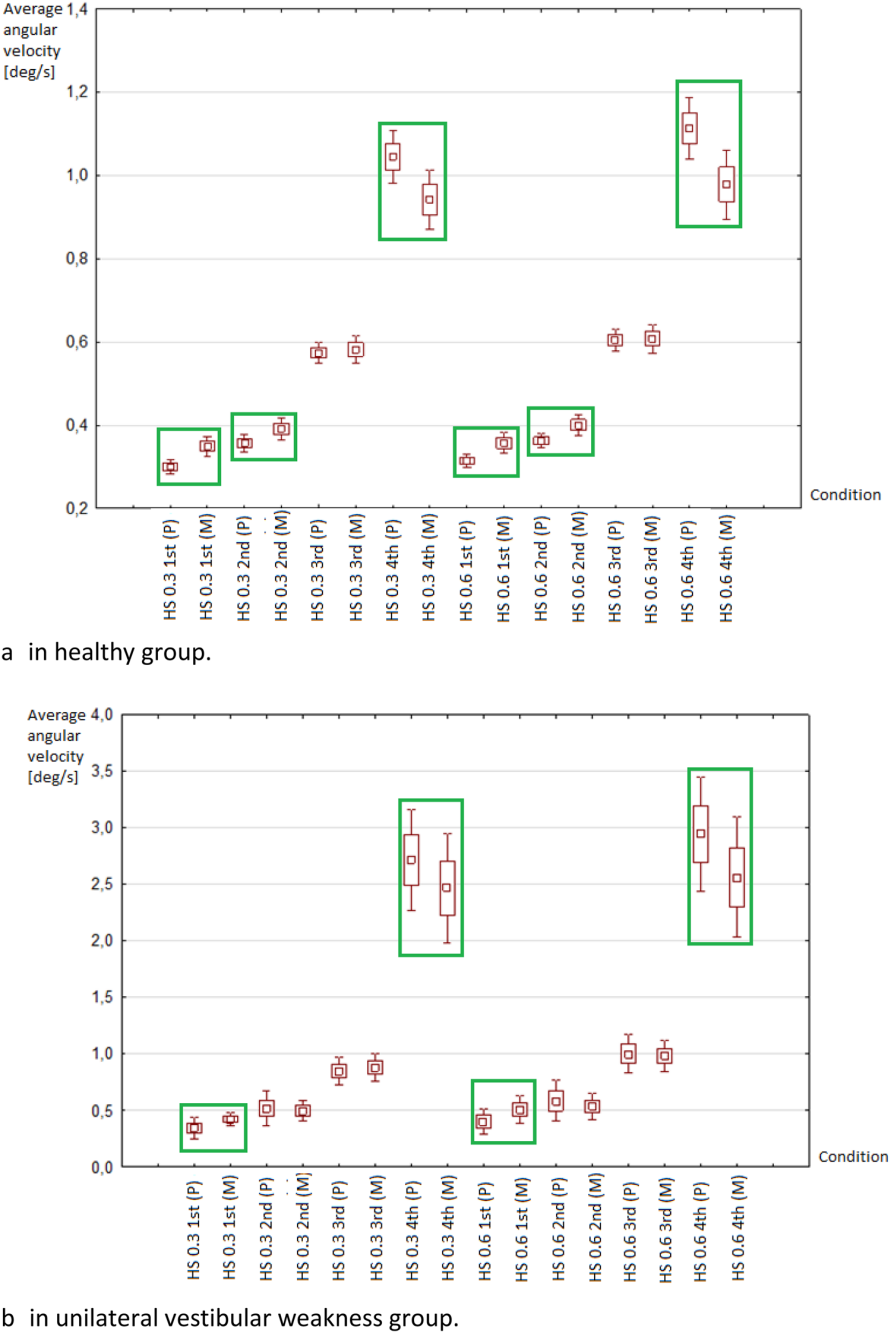
Distribution of average angular velocity values of the posturographic platform (P) and MediPost (M). HS 0.3—posturography with head frequency movements 0.3 Hz, HS 0.6—posturography with head movements at a frequency of 0.6 Hz; Conditions: 1st (eyes open/stable surface), 2nd (eyes closed/stable surface), 3rd (eyes open/foam), 4th (eyes closed/foam); *P* posturography platform, *M* MediPost (accelerometer device). Green boxes mark the statistically different mean values between the force plate and MediPost (p < 0.05).

The mean values of sway velocity in healthy groups were significantly higher for the MediPost as compared to the platform posturography for 1st and 2nd conditions in both HS 0.3 and 0.6 Hz tests. There were no differences between the devices’ results in the 3^rd^ condition, while in the 4th condition MediPost mean results were lower than platform results (Fig. 3a).

In the group with unilateral vestibular weakness the differences between devices were observed in the 1st and 4th conditions for both HS 0.3 and 0.6 Hz (Fig. 3b).

2. The correlations and the coefficient of agreement between the MediPost posturography results and the platform, based on the angular velocity.

**Figure 4:**
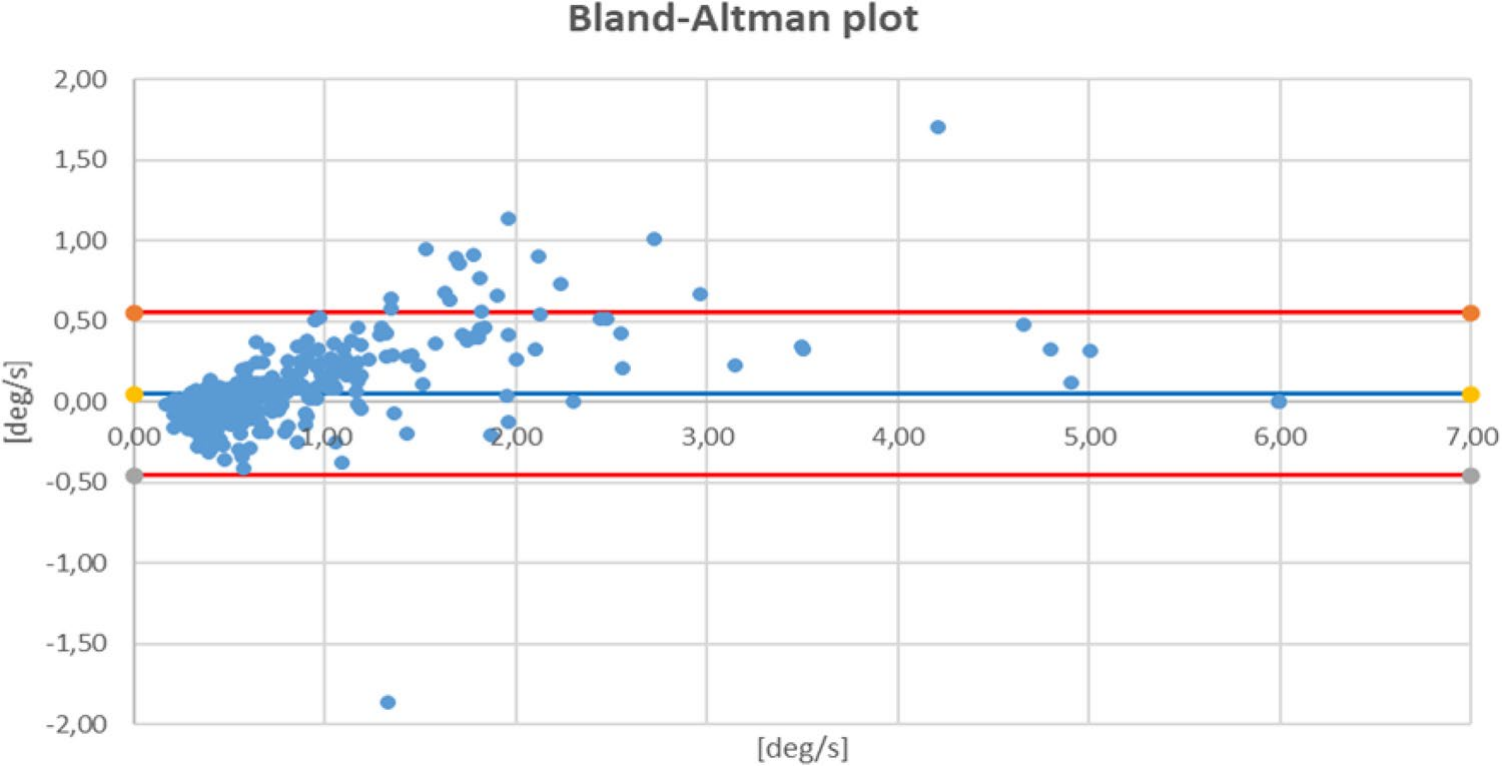
Bland–Altman plot for MediPost in relation to platform posturography results all subjects, lines represent differences expressed in terms of standard deviation (SD): red line: + 1.96 SD and − 1.96 SD.

The Bland–Altman plot gives a good coefficient consistency (96%) of the results of the compared methods (Fig. 4), which is in line with the Bland–Altman original study, where 95% agreement was quoted as the threshold^17^.

The Spearman correlation coefficients results were moderate (0.6 Hz 1st: 0. 6 and 0.6 Hz 2nd: 0.61); strong (0.3 Hz 1st: 0.76 and 0.3 Hz 2nd: 0.89) and very strong (0.3 Hz 3rd : 0.91 and 4th : 0.97; 0.6 Hz 3rd: 0.93 and 4th: 0.98).

3. The comparison of the sensitivity and specificity of the MediPost and the platform posturography.

The sensitivity and specificity were calculated and compared between methods. There were no statistically significant differences with p < 0.05 between the MediPost and the force plate for any condition in the 0.3 and 0.6 Hz tests. In both tests the highest values of the AUC were observed in the 4th condition (Table 1). In that condition the highest specificity (96%) with a high sensitivity (87% for MediPost and 84% for the force plate) were observed for 0.3 Hz test. In the 0.6 Hz test, MediPost revealed the highest specificity (88%) but lower sensitivity (82%) than the force plate.

**Table 1:** Sensitivity, specificity (the optimum cut-off point for the maximum value of Youden's index), areas under the receiver operating characteristic (ROC) in simultaneous measurements on the platform posturography (P) and the inertial sensor (M) attached to the lumbar L4.

Condition	Measurement method	AUC	p	Sensitivity	Specificity	PPV	NPV	Cut off point
Head shaking posturography 0.3 Hz								
1st	P	0.68	<.001	76	60	53	81	0.3
	M	0.64	0.01	92	43	49	90	0.3
2nd	P	0.73	<.001	55	80	62	75	0.4
	M	0.64	0.01	50	80	60	73	0.5
3rd	P	0.82	<.001	66	82	68	80	0.7
	M	0.82	<.001	74	82	70	84	0.7
4th	**P**	**0.94**	<.001	**84**	**95**	**91**	**91**	**1.9**
	**M**	**0.95**	<.001	**87**	**96**	**92**	**93**	**1.5**
Head shaking posturography 0.6 Hz								
1st	P	0.72	<.001	55	79	60	75	0.3
	M	0.70	<.001	58	79	61	76	0.4
2nd	P	0.78	<.001	63	86	73	80	0.4
	M	0.68	0.001	66	74	60	79	0.4
3rd	P	0.85	<.001	71	88	77	84	0.8
	M	0.87	<.001	74	92	85	86	0.8
4th	**P**	**0.92**	<.001	**95**	**77**	**70**	**96**	**1.5**
	**M**	**0.92**	<.001	**82**	**88**	**80**	**89**	**1.3**

Conditions: 1st (eyes open/stable surface), 2nd (eyes closed/stable surface), 3rd (eyes open/foam), 4th (eyes closed/foam).

The most significant results are in bold.

*P* platform posturography, *M* MediPost (IMU), *AUC* area under curve, *PPV* positive predictive value, *NPV* negative predictive value.

The additional test parameters (path length and surface) were presented for the MediPost only (Table 2). The specificity of these parameters was adjusted to the velocity specificity. Then the sensitivity was compared between the test parameters. For 0.3 Hz there were no improvements of sensitivity when comparing the path length and the surface parameters to the sway velocity parameter. For 0.6 Hz, the surface parameter revealed higher sensitivity in the 4th condition.

**Table 2:** The sensitivity and specificity of MediPost according to the measuring parameters. For comparison purposes the results of specificity for length and surface were adjusted to the value obtained for sway velocity.

Condition	Measurement parameter	AUC	p	Sensitivity	Specificity	PPV	NPV	Difference between sensitivity
**Head shaking posturography 0.3 Hz**								
1st	Velocity	0.64	0.01	92	43	48	90	0.0%
	Length	0.65	0.006	90	43	47	88	2.9%
	Surface	0.69	<.001	92	43	48	90	0.0%
2nd	Velocity	0.64	0.01	50	80	60	73	0.0%
	Length	0.65	0.01	47	80	58	73	5.3%
	Surface	0.62	0.009	34	80	52	68	31.6%
3rd	Velocity	0.82	<.001	74	82	70	84	3.4%
	**Length**	**0.85**	<.001	**76**	**82**	**71**	**86**	0.0%
	Surface	0.85	<.001	68	82	68	82	10.3%
4th	**Velocity**	**0.95**	<.001	**87**	**96**	**92**	**93**	0.0%
	**Length**	**0.96**	<.001	**87**	**96**	**92**	**93**	0.0%
	Surface	0.94	<.001	79	96	91	89	9.1%
**Head shaking posturography 0.6 Hz**								
1st	Velocity	0.70	0.001	58	79	61	76	0.0%
	Length	0.69	<.001	50	79	58	73	9.5%
	Surface	0.69	0.001	45	79	55	71	19.0%
2nd	Velocity	0.68	0.001	66	74	60	79	0.0%
	Length	0.69	<.001	58	74	56	75	12.0%
	Surface	0.67	0.003	50	74	53	72	24.%
3rd	Velocity	0.87	<.001	74	92	85	86	0.0%
	Length	0.90	<.001	72	92	84	85	3.6%
	Surface	0.89	<.001	68	92	84	84	7.1%
4th	Velocity	0.92	<.001	82	88	80	89	6.1%
	Length	0.93	<.001	82	88	80	89	6.1%
	**Surface**	**0.93**	<.001	**87**	**88**	**81**	**92**	0.0%

Conditions: 1st (eyes open/stable surface), 2nd (eyes closed/stable surface), 3rd (eyes open/foam), 4th (eyes closed/foam).

The most significant results are in bold.

*AUC* area under curve, *PPV* positive predictive value, *NPV* negative predictive value.

## Discussion

The aim of this study was to compare the results of the head shaking posturography performed according to the same tasks protocol on the MediPost IMU device and the standard force platform posturography. The main concern is how the differences between the devices, if present, are clinically relevant in patients with unilateral vestibular weakness. The second point of interest being whether the sway velocity is the most appropriate measuring parameter.

We found that comparing the two measuring devices, the mean values of sway velocities differ in both the healthy and the vestibular participant groups. For the 1st condition the mean results were significantly higher for the MediPost while in the 4th condition the mean values were lower.

On the contrary, the 4th condition (on the foam with eyes closed) is the “most difficult” one in the modified Clinical Test of Sensory Interaction on Balance (mCTSIB). The mean values were significantly lower for the MediPost measurements. The differences between the centre of pressure (COP) and the centre of mass (COM) registering may explain the lower MediPost values. The most common posturographic measure is the centre of pressure (COP), which is a vector sum of the ground reaction forces applied to the centre of mass (COM). Inertial based posturography directly determines the COM movements by mounting sensors on the L4 level. The COP-COM error was observed in the Choi and Kim study^18^. In that comparative study involving 3D motion analysis system, the COP sway was slightly larger than the COM in healthy volunteers. The COP-COM difference has been reported as an error of postural control system, which was correlated to whole body acceleration^19^. Corriveau recounted that the COP-COM difference is particularly great in older patients with neurological problems, meaning that the COP-COM difference increases with higher sways^20^. Similarly, the highest correlation coefficients between the MediPost and the force plate were observed in the vestibular patients’ group for the 4th condition in the test with fast head movements, which is where the sways were the highest.

If the body were to be thought of as moving like an inverted pendulum, a correlation close to 1 would be expected. In our study correlation coefficients close to 1 were observed in the vestibular group for the 0.3 Hz 4th condition and the 0.6 Hz 2nd and 4th conditions. Moreover, the high consistency of the results for both methods was demonstrated using the Bland–Altman plot. High correlation coefficients between the dynamic posturography and accelerometer’s posturography were observed by Whitney et al. in tests without the head movements. Similarly, to our research, the greatest correlations were observed for the more difficult conditions (4th, 5th, 6th of SOT protocol)^9^. No comparable studies were found for the head-shaking posturography so far.

Summarizing, the comparison of the two devices revealed differences between the COP and COM measurements. The main concern is how the differences listed above are clinically relevant. The second being, whether the sway velocity is the most appropriate measuring parameter.

To answer those questions, the sensitivity and specificity of the tests performed simultaneously on the platform and the MediPost were compared. The studies were performed on a group of people who had balance problems confirmed by the questionnaires and the dynamic posturography and a diagnosed dysfunction of the peripheral part of the vestibular system, regardless of the disease entity. The lack of homogeneity of the group could affect the sensitivity and specificity of the head shaking posturography, but should not influence the comparison of the two measurement devices.

The 4th condition on foam with eyes closed is the most relevant for vestibular assessment^21–23^. In the 4th condition, the cut-off points calculated from the ROC curves revealed the same pattern as the mean values. The cut-off points were lower for the MediPost than for the force plate posturography both in the 0.3 and 0.6 Hz head shaking tests. Despite the lower values, there were no statistically significant differences of the AUC between MediPost and the force plate in any of the tests. In the 0.3 Hz HS posturography both devices showed the same specificity, while the sensitivity was slightly higher for the MediPost. In the case of higher frequency of head movements (0.6 Hz) the MediPost measurements were more specific but less sensitive in revealing a vestibular impairment, than the force plate posturography.

Although the sensitivity and specificity calculations are not the main goal of our study, the highest sensitivity and specificity were observed for the 0.3 Hz head movement test, which is similar to our previous findings^6^. Moreover, the HS posturography results were better than for the standard static posturography. In the 4th condition of the HS posturography we obtained a sensitivity of 87% and specificity of 96%, while in the standard static posturography Di Fabio found 53% and 90% and El-Kashlan et al. 60% and 87%, respectively^3,24^. Our results were also markedly better than in the Cohen’s et al. study for the CTSiB protocol with head movement, in which the timing sensitivity was 71–81% and the specificity was 61–78%^5^. This might indicate that the sway velocity is a more valuable parameter than the amount of time standing without falling.

In our static platform posturography, we only had results of the angular velocity of the sways. Other posturography studies typically used one of the many parameters which could be evaluated to compare the accelerometer and the force plate. For instance, in the Whitney study, the highest correlations were observed for the normalized path length (0.88–0.96 for 6th condition of dynamic posturography), in the Mancini et al. study that was the centroidal frequency (0.89) while Alessandrini et al. found the correlation coefficients close to 1 for the distance of the COP or COM and the measurements of characteristic frequencies in the power spectral density of the sways^10,11^. Such highly sophisticated parameters are usually not a subject in a clinical evaluation.

## Conclusions

The MediPost IMU device and the force platform posturography revealed a similar ability to differentiate between the patients with balance problems in the course of vestibular pathology and the healthy participants, despite the differences observed between measuring methods. Thus, the MediPost seems to be a good alternative to force plate posturography. In the test with the slow head movements, the sensitivity of MediPost seems to be slightly higher than the force plate. In the test with higher frequency of head movements, surface parameter may be more appropriate than sway velocity in order to improve MediPost’s sensitivity.

## References

[1] Shepard, N.T., & Speers, R.A. Head Movement Modification to Sensory Organization Protocol of EquiTest: Clinical Utility in a Random Sample of Balance Disorder Patients*.* in *20’th Barany Society Meeting, Wuerzburg, Germany* (1998).

[2] Honaker JA, Janky KL, Patterson JN, Shepard NT (2016). Modified head shake sensory organization test: Sensitivity and specificity. Gait Posture..

[3] Di Fabio RP (1995). Sensitivity and specificity of platform posturography for identifying patients with vestibular dysfunction. Phys Ther..

[4] Cohen HS, Mulavara AP, Peters BT, Haghpeykar HS, Bloomberg JJ (2014). Standing balance tests for screening people with vestibular impairments. Laryngoscope.

[5] Cohen HS, Mulavara AP, Stitz J (2019). Screening for vestibular disorders using the modified clinical test of sensory interaction and balance and tandem walking with eyes closed. Otol. Neurotol..

[6] Janc M, Sliwinska-Kowalska M, Politanski P (2021). Posturography with head movements in the assessment of balance in chronic unilateral vestibular lesions. Sci. Rep..

[7] Ghislieri M, Gastaldi L, Pastorelli S, Tadano S, Agostini V (2019). Wearable inertial sensors to assess standing balance: A systematic review. Sensors (Basel).

[8] Johnston W, O’Reilly M, Argent R, Caulfield B (2019). Reliability, validity and utility of inertial sensor systems for postural control assessment in sport science and medicine applications: A systematic review. Sports Med..

[9] Whitney SL, Roche JL, Marchetti GF (2011). A comparison of accelerometry and center of pressure measures during computerized dynamic posturography: A measure of balance. Gait Posture..

[10] Mancini M, Salarian A, Carlson-Kuhta P (2012). ISway: A sensitive, valid and reliable measure of postural control. J. NeuroEng. Rehabil..

[11] Alessandrini M, Micarelli A, Viziano A (2017). Body-worn triaxial accelerometer coherence and reliability related to static posturography in unilateral vestibular failure. Acta Otorhinolaryngol. Ital..

[12] (ICVD) International Classification of Vestibular Disorders. http://www.jvr-web.org/Barany-feedback.html. (online).

[13] Allum JHJ, Scheltinga A, Honegger F (2017). The effect of peripheral vestibular recovery on improvements in vestibulo-ocular reflexes and balance control after acute unilateral peripheral vestibular loss. Otol. Neurotol..

[14] Lopez-Escamez JA, Carey J, Chung WH (2015). Classification Committee of the Barany Society; Japan Society for Equilibrium Research; European Academy of Otology and Neurotology (EAONO); Equilibrium Committee of the American Academy of Otolaryngology-Head and Neck Surgery (AAO-HNS). J. Vestib. Res..

[15] Kotas R, Janc M, Kaminski M (2019). Evaluation of agreement between static posturography methods employing tensometers and inertial sensors. IEEE..

[16] Mukaka MM (2012). Statistics corner: A guide to appropriate use of correlation coefficient in medical research. Malawi Med J..

[17] Bland JM, Altman DG (1999). Measuring agreement in method comparison studies. Stat. Methods Med. Res..

[18] Choi, H.S., & Kim, Y.H. The relationship between the COP–COM variable and the horizontal acceleration of the body in postural sway, falling and walking*.* in Dössel O., Schlegel W.C. (eds) *World Congress on Medical Physics and Biomedical Engineering, September 7–12, 2009, Munich, Germany. IFMBE Proceedings*, Vol. 25/4. 10.1007/978-3-642-03882-2_74 (Springer, 2009).

[19] Morasso PG, Spada G, Capra R (1999). Computing the COM from the COP in postural sway movements. Hum. Mov. Sci..

[20] Corriveau H, Hébert R, Raîche M, Dubois MF, Prince F (2004). Postural stability in the elderly: Empirical confirmation of a theoretical model. Arch. Gerontol. Geriatr..

[21] Shumway-Cook A, Horak FB (1986). Assessing the influenceof sensory interaction on balance. Phys. Thm..

[22] Allum JH, Adkin AL, Carpenter MG, Held-Ziolkowska M, Honegger F, Pierchala K (2001). Trunk sway measures of postural stability during clinical balance tests: Effects of a unilateral vestibular deficit. Gait Posture..

[23] Allum JHJ, Zamani F, Adkin AL, Ernst A (2002). Differences between trunk sway characteristics on a foam support surface and on the Equitest ankle-sway-referenced support surface. Gait Posture.

[24] El-Kashlan HK, Shepard NT, Asher AM, Smith-Wheelock M, Telian SA (1998). Evaluation of clinical measures of equilibrium. Laryngoscope..

